# Uneven distribution of ventilation in acute respiratory distress syndrome

**DOI:** 10.1186/cc3058

**Published:** 2005-02-21

**Authors:** Christian Rylander, Ulf Tylén, Rauni Rossi-Norrlund, Peter Herrmann, Michael Quintel, Björn Bake

**Affiliations:** 1 Department of Anaesthesiology and Intensive Care, Sahlgrenska University Hospital, Göteborg, Sweden; 2 Professor, The Sahlgrenska Academy at Göteborg University, Department of Radiology, Sahlgrenska University Hospital, Göteborg, Sweden; 3 The Sahlgrenska Academy at Göteborg University, Department of Radiology, Sahlgrenska University Hospital, Göteborg, Sweden; 4 Engineer, Department of Anaesthesiology II – Intensive Care Medicine, Z.A.R.I., University Hospital Gottingen, Gottingen, Germany; 5 Professor, Department of Anaesthesiology II – Intensive Care Medicine, Z.A.R.I., University Hospital Gottingen, Gottingen, Germany; 6 Professor, The Sahlgrenska Academy at Göteborg University, Department of Pulmonary Medicine, Sahlgrenska University Hospital, Göteborg, Sweden

## Abstract

**Introduction:**

The aim of this study was to assess the volume of gas being poorly ventilated or non-ventilated within the lungs of patients treated with mechanical ventilation and suffering from acute respiratory distress syndrome (ARDS).

**Methods:**

A prospective, descriptive study was performed of 25 sedated and paralysed ARDS patients, mechanically ventilated with a positive end-expiratory pressure (PEEP) of 5 cmH_2_O in a multidisciplinary intensive care unit of a tertiary university hospital. The volume of poorly ventilated or non-ventilated gas was assumed to correspond to a difference between the ventilated gas volume, determined as the end-expiratory lung volume by rebreathing of sulphur hexafluoride (EELV_SF6_), and the total gas volume, calculated from computed tomography images in the end-expiratory position (EELV_CT_). The methods used were validated by similar measurements in 20 healthy subjects in whom no poorly ventilated or non-ventilated gas is expected to be found.

**Results:**

EELV_SF6 _was 66% of EELV_CT_, corresponding to a mean difference of 0.71 litre. EELV_SF6 _and EELV_CT _were significantly correlated (*r*^2 ^= 0.72; *P *< 0.001). In the healthy subjects, the two methods yielded almost identical results.

**Conclusion:**

About one-third of the total pulmonary gas volume seems poorly ventilated or non-ventilated in sedated and paralysed ARDS patients when mechanically ventilated with a PEEP of 5 cmH_2_O. Uneven distribution of ventilation due to airway closure and/or obstruction is likely to be involved.

## Introduction

Decreased functional residual capacity (FRC) and increased pulmonary resistance are hallmarks of acute respiratory distress syndrome (ARDS) [1]. Pathophysiological mechanisms include alveolar flooding and/or collapse, which contribute to shunting of blood and to hypoxaemia [2]. Whether true alveolar collapse or intraluminar oedema with increased impedance dominates is a matter of debate [3]. Furthermore, the expiratory flow limitation observed in ARDS patients has been attributed to the closure of small airways [4]. Pulmonary gas distal to such an airway closure/obstruction may be poorly ventilated or non-ventilated. If so, it might not be included in FRC measurements based on tracer gas dilution. The end-expiratory lung volume determined by tracer gas dilution is termed 'ventilated gas volume' in this paper. Other techniques such as body plethysmography and radiographical methods [5] determine the total end-expiratory volume of pulmonary gas, irrespective of whether it is well ventilated, poorly ventilated or non-ventilated. This volume is termed 'total gas volume' in this report. A difference between the ventilated gas volume and the total gas volume can be interpreted as a volume of gas being poorly ventilated or non-ventilated. This difference is obvious in patients with chronic obstructive airway disease in whom FRC determined by gas dilution might be considerably lower than FRC determined by body plethysmography [6]. However, in mechanically ventilated ARDS patients the volume of poorly ventilated or non-ventilated gas seems not to have been studied in detail.

The aim of the present study was therefore to assess the volume of poorly ventilated or non-ventilated gas in mechanically ventilated ARDS patients, assuming the difference between the ventilated gas volume and the total gas volume to represent poorly ventilated or non-ventilated gas. To validate the methods involved, similar measurements were performed in young healthy subjects in whom no poorly ventilated or non-ventilated gas is expected to be found.

## Materials and methods

### Ethical approval

The study was approved by the local ethics committee and conducted in accordance with the Helsinki Declaration. Informed consent was obtained from the next-of-kin of the patients and directly from the healthy subjects.

### Patients

Twenty-five sedated and mechanically ventilated patients were included from a mixed-adult intensive care unit. The criterion for selection was the eligible ARDS patient [7] having spent the longest time on mechanical ventilation at the time of the once-weekly available opportunity for computed tomography (CT). Patients were eligible for the study only if their arterial oxygenation was stable and between 10 and 26 kPa during mechanical ventilation with the following parameters: fraction of inspired oxygen 0.5; constant flow volume-controlled mode; tidal volume 8 to 10 ml/kg; positive end-expiratory pressure (PEEP) 5 cmH_2_O. Chronic obstructive pulmonary disease was not an exclusion criterion but was present only in one patient (no. 13). Clinical data are given in Table 1. Twenty healthy non-smoking students independent of the investigating institutions were enrolled and interviewed to rule out any history of tobacco use or obstructive lung disease. Anthropometric data for both groups are given in Table 2.

### Measurements

**Figure 1:**
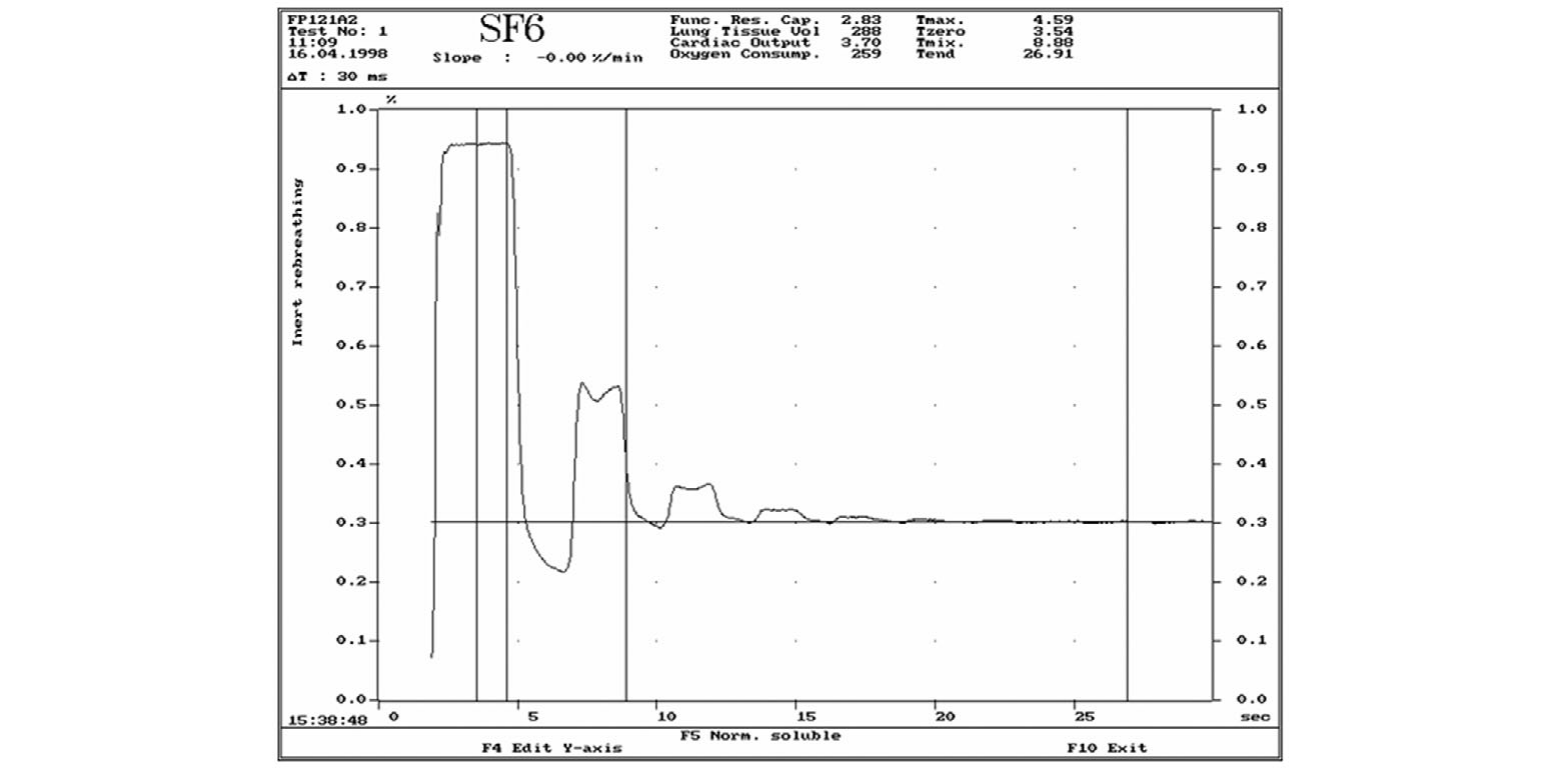
Concentration of the tracer gas sulphur hexafluoride (SF_6_) plotted during 30 s of rebreathing in a supine healthy subject.

The ventilated gas volume was determined in both groups by a gas dilution technique using rebreathing of sulphur hexafluoride. End-expiratory measurements in the ventilated patients were made at a PEEP of 5 cmH_2_O (EELV_SF6_) and measurements in the spontaneously breathing healthy subjects were made at the FRC level (FRC_SF6_). A prototype system (AMIS 2001; Innovision A/S, Odense, Denmark) equipped with a photoacoustic and magnetoacoustic multigas analyser [8] was used. The accuracy of the analyser was checked by comparison with mass spectrometry (AMIS 2000; Innovision A/S) before and after the series of experiments. Before each measurement, the ambient temperature and pressure were registered and correct readings from the gas analyser were verified by supplying room air and the undiluted tracer gas mixture to the gas inlet. The gas sampling rate was 120 ml/min. The rebreathing unit consisted of a bag-in-box system in which the flexible rubber bellows could be manually ventilated by a piston fitted through the distal short end of the cylinder. For operation, the unit was instantly switched into the patient circuit by a pneumatic slide valve without disconnection. The bellows was initially filled with 1.2 litres (ambient temperature and pressure, dry) of a gas mixture of 1.0% SF_6 _in 5.0% nitrous oxide (N_2_O) and oxygen (bal; medical grade). The presence of N_2_O was due to the circulatory monitoring function of the multimodal monitoring system. The SF_6 _concentration was continuously plotted during 30 s of ventilation at a frequency of 20 breaths per minute (Fig. 1). Allowing for the tubing dead space (101 ml in the subjects, 107 ml in the patients), the ventilated gas volume was calculated from a formula based on standard gas dilution principles for FRC measurements:



where *P*_b _is the barometric pressure in torr, *T *is the ambient absolute temperature and SF_6i _and SF_6e _are the initial and equilibrated concentration of SF_6 _(standard temperature and pressure, dry), respectively, and 1.2 is the bellows volume. FRC symbolises both FRC_SF6 _in the young healthy subjects and EELV_SF6 _in the ventilated patients.

The total gas volume was calculated from CT images reconstructed from a scan lasting about 20 s in a high-speed scanner (GE High Speed CT/i; General Electric Medical Systems, Milwaukee, WI, USA). End-expiratory measurements in the patients were made in apnoea at PEEP 5 cmH_2_O (EELV_CT_) and measurements in the healthy subjects were made in apnoea at the FRC level (FRC_CT_). The following exposure parameters were used: 120 kV; 170 mA; rotation time 1.0 s; collimation 1 mm and a matrix of 512 × 512, yielding voxel volumes of 0.25 to 0.49 mm^3 ^depending on the field of view. An initial topogram defined the limits of the lungs, and the first and last scanning levels were positioned at the apical and caudal extremes, respectively. In between, eight more scanning levels were evenly dispersed, making a total of 10 consecutive single exposures with a distance between the scans of 18 to 25 mm, depending on thoracic dimensions. The total effective radiation dose was estimated to equal one standard chest X-ray examination, yielding an average absorbed radiation of 0.8 mGy to the breasts of female subjects. Within each image, the lungs were manually delineated from the thoracic wall in a single region of interest. Within the region of interest, the voxels with attenuation values between – 1,000 and 0 Hounsfield units (HU) were automatically selected for analysis by software (MALUNA 2.02; Peter Herrmann, Mannheim, Germany) on a personal computer, and their gas volume (*V*) was calculated from the formula [9]



where *V*_vox _is the single-voxel volume of *n *voxels within the slice. The total gas volume was calculated by interpolating for the volume of gas in the lung tissue between the 10 scan levels by the method of Kvist [10] with the modified formula



where *V*_1 _and *V*_2 _are the gas volumes of two adjacent slices with the thickness *t*, separated by the centre distance *d*. FRC symbolises both FRC_CT _in the young healthy subjects and EELV_CT _in the ventilated patients.

During the measurements, the sedated patients were temporarily paralysed and ventilated by means of a mobile ventilator (Servo 900 C; Siemens, Solna, Sweden) with the settings described above. The end-expiratory position was achieved by activation of the expiratory hold function on the ventilator. The patient was then either ventilated from the rebreathing circuit or CT scanned in maintained apnoea. The rebreathing procedure was performed in duplicate before and after a single CT exposure.

Before the supine measurements, the nose-clipped, supine and relaxed healthy subjects breathed room air through a mouthpiece connected to the rebreathing system through a three-way valve. At the FRC level, the valve was either switched into the rebreathing system for gas dilution by spontaneous breathing or was closed during the CT examination. The rebreathing procedure was performed in duplicate before and after a single CT exposure.

### Statistical analysis

Data are presented as means ± standard deviation if not specified otherwise. The level of significance was defined as *P *< 0.05. The coefficient of variation (CV) for paired measurements was calculated as the standard deviation of the differences divided by the mean of all measurements [11]. Calculations were performed with the software package Statistica 6.0 (StatSoft Inc., Tulsa, OK, USA) on a personal computer.

## Results

**Figure 2:**
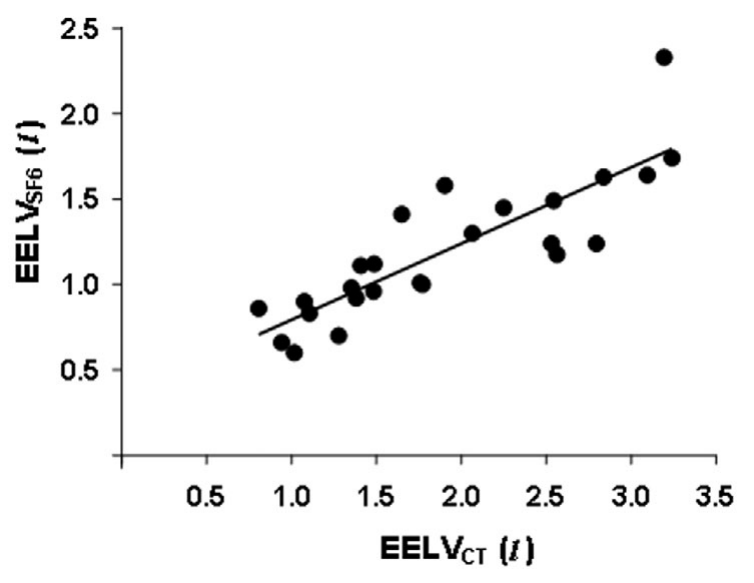
Linear regression between EELV measurements by rebreathing of sulphur hexafluoride (EELV_SF6_) and by computed tomography (EELV_CT_) obtained in 25 ARDS patients. The dotted line is the regression line EELV_SF6 _= 0.4EELV_CT _+ 0.3 (*r*^2 ^= 0.72; *P *< 0.001).

In the ARDS patients, EELV_SF6 _was 66 ± 14% of EELV_CT_. EELV_SF6 _was found systematically lower than EELV_CT _except in one patient (no. 19), in whom they were similar. The mean difference, corresponding to the poorly ventilated or non-ventilated gas volume, was 0.71 ± 0.47 litre. The magnitude of the poorly ventilated or non-ventilated gas volume was not correlated with age or ventilator days. Mean results are given in Table 3. EELV_SF6 _and EELV_CT _were significantly correlated (*r *= 0.85; *P *< 0.001) (Fig. 2). The CV of duplicate EELV_SF6 _measurements was 5.6%.

**Figure 3:**
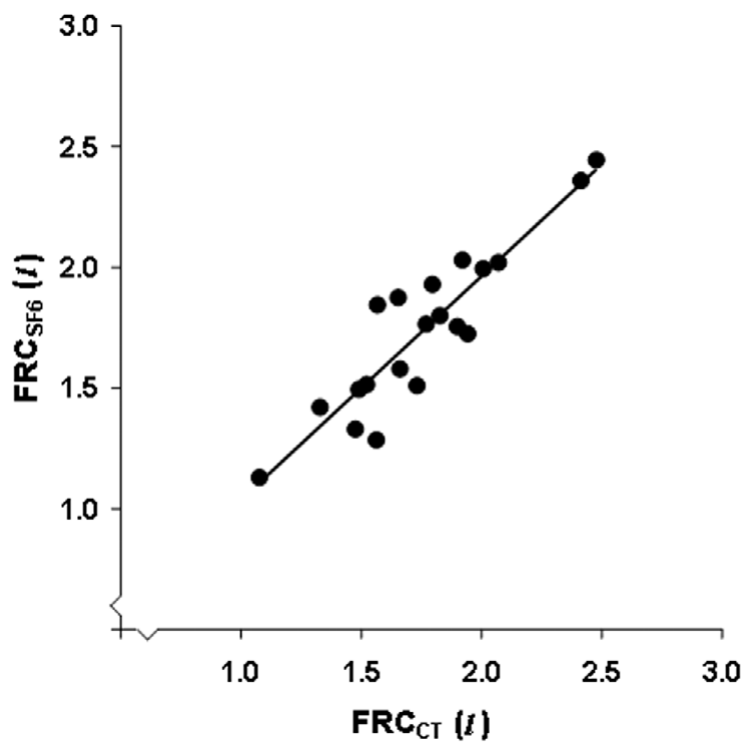
Linear regression between FRC measurements by rebreathing of sulphur hexafluoride (FRC_SF6_) and by computed tomography (FRC_CT_) in 20 healthy subjects. The dotted line is the regression line: EELV_SF6 _= 0.9FRC_CT _+ 0.1 (*r*^2 ^= 0.83; *P *< 0.001).

**Figure 4:**
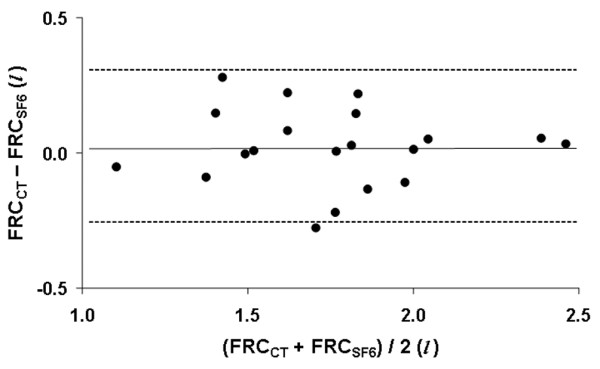
Bland-Altman plot [24] of supine functional residual capacity measured by rebreathing of sulphur hexafluoride (FRC_SF6_) and by computed tomography (FRC_CT_) in 20 healthy subjects. The individual differences of paired measurements (*y *axis) did not depend on the magnitude of their average values (*x *axis). The mean difference (solid line; dotted lines represent the mean ± 2SD) was small.

In the supine healthy subjects, FRC_SF6 _was 99 ± 9% of FRC_CT_, and they were closely correlated (*r *= 0.91; *P *< 0.001) (Fig. 3). The differences did not depend on the magnitude of FRC (Fig. 4). The CV of duplicate FRC_SF6 _measurements was 3.1%.

## Discussion

This study shows that there is a considerable volume of poorly ventilated or non-ventilated gas present in the lungs of sedated and paralysed ARDS patients when mechanically ventilated with a PEEP of 5 cmH_2_O.

We assumed that the difference between the ventilated gas volume determined by gas dilution and the total gas volume calculated from CT corresponds to a poorly ventilated or non-ventilated gas volume. The methods used to determine these volumes were validated by comparison of similar measurements in young healthy subjects, in whom they should yield similar results because these lungs are homogeneously ventilated with no obstruction and no airway closure. Indeed, almost identical results were obtained in the young healthy subjects. Furthermore, the CV of duplicate measurements in the healthy subjects indicated a good repeatability. The FRC_SF6 _values might seem somewhat low compared with predicted FRC values based on a mixed adult population (Table 3), but normal FRC values in supine young subjects are rare and the predictions therefore remain uncertain. The CT interpolation technique has been validated previously for heterogeneously scattered tissue [12] and should be precise enough with 10 scans evenly distributed over the lungs. In summary, we consider that the two methods used were adequate and that the difference between their results in the ARDS patients can be assumed to correspond to a poorly ventilated or non-ventilated gas volume.

The most likely pathophysiological mechanism associated with this volume is airway closure and/or obstruction. Further contribution from atelectasis formation during the inspiratory hold is unlikely with the fraction of inspiratory oxygen used [13]. However, the deep sedation and paralysis of the ARDS patients might have contributed to poor ventilation in the dependent parts of the lungs [14]. The lung injury is unevenly distributed in ARDS [15], which causes an uneven distribution of ventilation including overdistension of non-dependent regions. By definition, open but non-compliant lung units are poorly ventilated or non-ventilated, but this seems unlikely to be of any importance in ARDS patients during ventilation with a PEEP of 5 cmH_2_O.

The PEEP level applied in the present study was chosen to be clinically relevant [16] but it does not effectively counteract expiratory derecruitment of lung units. In a study of 10 ARDS patients, mechanically ventilated with zero end-expiratory pressure (ZEEP), Koutsoukou and colleagues determined an intrinsic PEEP of 4.1 ± 2.4 cmH_2_O, and expiratory flow limitation was demonstrated in eight of them [4]. These results suggest the presence of airway closure and/or obstruction at the FRC level in ARDS. In contrast, when closed circuit helium rebreathing and CT were recently compared in a group of 21 ARDS patients, mechanically ventilated with a PEEP of 12 ± 5 cmH_2_O, similar EELVs were found [17]. This finding indicates that there is no airway closure and/or obstruction when a PEEP of 12 cmH_2_O is applied. Indeed, it was recently also shown that the intrinsic PEEP and the expiratory flow limitation present at ZEEP can be eliminated by a PEEP of 10 cmH_2_O [18]. In summary, those studies and the present results indicate that airway closure and/or obstruction occurs at low levels of PEEP or ZEEP and that the distal gas volume is recruitable for more effective ventilation by a moderate increase in PEEP. Accordingly, increasing PEEP from 0 to 15 cmH_2_O has been shown in a study of pulmonary mechanics to increase pulmonary compliance in some patients, which was associated with the recruitment of lung units with preserved normal compliance [19]. Furthermore, low compliance during the initial phase of inspiration has been attributed to non-collapsed but slowly ventilated lung units, in which the ventilation can be increased by increased PEEP [20]. The gas content of such non-collapsed but poorly ventilated lung units may correspond to the volume of poorly ventilated or non-ventilated gas demonstrated in the present study.

Substantially elevated pressure in the airways is associated with signs of parenchymal overdistension [21]. CT studies have shown that this effect is located to non-dependent well-aerated lung units that become overdistended by the airway pressure required to inflate compressed dependent lung units [22]. Overdistension associated with increased airway pressure seems to be less pronounced when the parenchyma is diffusely affected without regional atelectasis [23], as in our patients. Possibly, the poorly ventilated or non-ventilated gas volume in this type of diffuse ARDS might reflect gas contained in lung units distal to airway closure and/or obstruction. The recruitment of such gas-containing lung units, excluded from effective ventilation by partial compression or oedema, can be expected to require a smaller elevation of transmural pressure than that needed to inflate completely collapsed lung units. If the volume of poorly ventilated or non-ventilated gas is small or non-existent, a moderately raised airway pressure might be ineffective for recruitment and merely contribute to the risk of overdistension.

## Conclusion

We conclude that about one-third of the total gas volume is poorly ventilated or non-ventilated in the lungs of sedated and paralysed ARDS patients when mechanically ventilated with a PEEP of 5 cmH_2_O. This indicates uneven distribution of ventilation due to the presence of small-airway closure and/or obstruction at this PEEP level. Such a poorly ventilated or non-ventilated gas volume might be recruited for more effective ventilation by an increase in airway pressure that is less than the inflation pressure of completely collapsed lung units.

## Key messages

• 	This study demonstrates uneven distribution of ventilation in 25 sedated and ventilated ARDS patients by comparing the total end-expiratory gas end volume calculated from computed tomography and the ventilated gas volume measured by inert gas rebreathing.

• 	The poorly ventilated or non-ventilated volume distal to the possible airway closure and/or obstruction might be recruited for more effective ventilation by an increase in airway pressure that is less than the inflation pressure of completely collapsed lung units.

## Abbreviations

ARDS = acute respiratory distress syndrome; CT = computed tomography; CV = coefficient of variation; EELV_CT _= total gas volume calculated from computed tomography images in the end-expiratory position; EELV_SF6 _= end-expiratory lung volume measured by rebreathing of sulphur hexafluoride; FRC = functional residual capacity; FRC_CT _= FRC calculated from computed tomography scans; FRC_SF6 _= FRC measured by sulfur hexafluoride rebreathing; HU = Hounsfield unit; PEEP = positive end-expiratory pressure; ZEEP = zero end-expiratory pressure.

## Competing interests

The author(s) declare that they have no competing interests.

##   Authors' contributions

CR, UT and BB conceived the study and designed the protocol. UT, MQ and PH defined the radiographical image analysis. CR and RRN performed measurements. CR, UT and BB wrote and revised the manuscript, which was reviewed and approved by all authors before final submission.

## Figures and Tables

**Table 1:** Clinical data of the ARDS patients

Patient	Age (years)	Diagnosis	Ventilator days	Discharge status
1	52	Bilateral pneumonia	4	S
2	44	Sepsis with MOF	63	S
3	79	Postoperative MOF	10	NS
4	37	Bilateral pneumonia	40	S
5	85	Postoperative MOF	3	NS
6	18	Postoperative ARDS	2	S
7	54	Bilateral pneumonia	5	S
8	79	Postoperative ARDS	2	S
9	62	Bilateral pneumonia	5	NS
10	60	Aspiration	3	NS
11	46	Postoperative ARDS	3	S
12	44	Bilateral pneumonia	6	S
13	76	Bilateral pneumonia	13	S
14	22	Multiple trauma	2	S
15	78	Bilateral pneumonia	4	NS
16	58	Postoperative sepsis	3	NS
17	29	Multiple trauma	10	S
18	63	Pneumonia	4	S
19	53	Postoperative sepsis	26	S
20	71	Postoperative ARDS	2	S
21	31	Multiple trauma	3	S
22	22	Multiple trauma	5	S
23	70	Bilateral pneumonia	12	NS
24	82	Bilateral pneumonia	13	NS
25	20	Bilateral pneumonia	7	S

Mean	53	-	10	8/25 NS

Ventilator days were calculated on the day of study. ARDS, acute respiratory distress syndrome; MOF, multi-organ failure; NS, non-survivor; S, survivor.

**Table 2:** Anthropometric data

Group	*n*	Age (years)	Sex (M/F)	Height (cm)	BMI (kg/m^2^)
ARDS patients	25	53 (18–85)	13/12	174 (165–195)	25 (17–30)
Healthy subjects	20	24 (19–28)	8/12	173 (161–192)	22 (18–25)

Data are given as mean and range except for number and gender. ARDS, acute respiratory distress syndrome; BMI, body mass index.

**Table 3:** Lung volumes

Group	Supine EELV or FRC (litres)	
	
	SF_6_	CT
ARDS patients	1.2 ± 0.4	1.9 ± 0.8
Healthy subjects	1.7 ± 0.3	1.8 ± 0.3
	(78% of predicted)	(80% of predicted)

End-expiratory lung volume (EELV) in the acute respiratory distress syndrome (ARDS) patients and functional residual capacity (FRC) in the healthy subjects were measured by rebreathing of sulphur hexafluoride (SF_6_) and computed tomography (CT), respectively. Predicted normal FRC values are from [25].
